# *Helicobacter pylori* ATCC 43629/NCTC 11639 Outer Membrane Vesicles (OMVs) from Biofilm and Planktonic Phase Associated with Extracellular DNA (eDNA)

**DOI:** 10.3389/fmicb.2015.01369

**Published:** 2015-12-16

**Authors:** Rossella Grande, Maria C. Di Marcantonio, Iole Robuffo, Arianna Pompilio, Christian Celia, Luisa Di Marzio, Donatella Paolino, Marilina Codagnone, Raffaella Muraro, Paul Stoodley, Luanne Hall-Stoodley, Gabriella Mincione

**Affiliations:** ^1^Department of Pharmacy, “G. d'Annunzio” University of Chieti-PescaraChieti, Italy; ^2^Center of Excellence on Aging, Ce.S.I., “G. d'Annunzio” University of Chieti-PescaraChieti, Italy; ^3^Department of Medical, Oral, and Biotechnological Sciences, “G. d'Annunzio” University of Chieti-PescaraChieti, Italy; ^4^Department of Biological Sciences, Institute of Molecular Genetics, National Research CouncilChieti, Italy; ^5^Department of Nanomedicine, Houston Methodist Research InstituteHouston, TX, USA; ^6^Department of Clinical and Experimental Medicine, University of Catanzaro “Magna Graecia”Catanzaro, Italy; ^7^Department of Microbial Infection and Immunity, Center for Microbial Interface Biology, The Ohio State UniversityColumbus, OH, USA; ^8^Department of Orthopaedics, The Ohio State UniversityColumbus, OH, USA; ^9^Faculty of Engineering and the Environment, University of SouthamptonSouthampton, UK; ^10^NIHR Wellcome Trust Clinical Research Facility, University Hospital Southampton NHS Foundation TrustSouthampton, UK

**Keywords:** *Helicobacter pylori*, eDNA, outer membrane vesicles (OMVs), biofilm, nanoparticles

## Abstract

*Helicobacter pylori* persistence is associated with its capacity to develop biofilms as a response to changing environmental conditions and stress. Extracellular DNA (eDNA) is a component of *H. pylori* biofilm matrix but the lack of DNase I activity supports the hypothesis that eDNA might be protected by other extracellular polymeric substances (EPS) and/or Outer Membrane Vesicles (OMVs), which bleb from the bacteria surface during growth. The aim of the present study was to both identify the eDNA presence on OMVs segregated from *H. pylori* ATCC 43629/NCTC 11639 biofilm (bOMVs) and its planktonic phase (pOMVs) and to characterize the physical-chemical properties of the OMVs. The presence of eDNA in bOMVs and pOMVs was initially carried out using DNase I-gold complex labeling and Transmission Electron Microscope analysis (TEM). bOMVs and pOMVs were further isolated and physical-chemical characterization carried out using dynamic light scattering (DLS) analysis. eDNA associated with OMVs was detected and quantified using a PicoGreen spectrophotometer assay, while its extraction was performed with a DNA Kit. TEM images showed that eDNA was mainly associated with the OMV membrane surfaces; while PicoGreen staining showed a four-fold increase of dsDNA in bOMVs compared with pOMVs. The eDNA extracted from OMVs was visualized using gel electrophoresis. DLS analysis indicated that both planktonic and biofilm *H. pylori* phenotypes generated vesicles, with a broad distribution of sizes on the nanometer scale. The DLS aggregation assay suggested that eDNA may play a role in the aggregation of OMVs, in the biofilm phenotype. Moreover, the eDNA associated with vesicle membrane may impede DNase I activity on *H. pylori* biofilms. These results suggest that OMVs derived from the *H. pylori* biofilm phenotype may play a structural role by preventing eDNA degradation by nucleases, providing a bridging function between eDNA strands on OMV surfaces and promoting aggregation.

## Introduction

*Helicobacter pylori* is capable of adapting itself to both the natural environment and to the human host (Mackay et al., 1998; Carron et al., 2006; García et al., 2014). However, the favored niche of bacterium is the gastric epithelium of the human stomach. *H. pylori* colonizes nearly 50% of individuals world-wide (García et al., 2014) and represents a major risk factor for developing gastritis, peptic ulcer disease, mucosa-associated lymphoid tissue lymphoma (MALT), and gastric cancer (Eshraghian, 2014). The clinical outcome of *H. pylori* infection depends on multiple factors, which modulate host-pathogen interactions (Fernandez-Gonzalez and Backert, 2014). In particular, *H. pylori* persistence and recalcitrance might be associated to both its broad genetic variability and capability of developing biofilm (Stark et al., 1999; Cole et al., 2004; Morelli et al., 2010).

Several studies have demonstrated that recombination events occurred frequently during chronic infections and generate multiple *H. pylori* mosaic genotypes (Kersulyte et al., 1999; Falush et al., 2001; Grande et al., 2010; Morelli et al., 2010). *H. pylori* also forms biofilms, complex structures characterized by cells embedded in a matrix of Extracellular Polymeric Substances (EPS), comprised of proteins, carbohydrates and DNA, which also may play a role in bacterial survival and persistence (Percival and Suleman, 2014). Furthermore, biofilms represent a protected environment promoting recombination events that may increase virulence and antimicrobial resistance (Grande et al., 2012).

Recently, several studies have described the role of deoxyribonucleic acid (DNA) as a major component of the biofilm extracellular matrix in both Gram-positive (Moscoso et al., 2006; Rice et al., 2007; Hall-Stoodley et al., 2008; Izano et al., 2008) and Gram-negative (Whitchurch et al., 2002; Allesen-Holm et al., 2006; Harmsen et al., 2010) bacteria. These studies demonstrated that eDNA plays a crucial role as a structural component of the biofilm extracellular matrix. In previous work, we showed that eDNA is a component of *H. pylori* EPS. However, the lack of DNase I activity in reducing the biofilm biomass suggested that eDNA might be associated with other EPS components (Grande et al., 2011).

Outer membrane vesicles (OMVs) have also been shown to be an important EPS component of *Pseudomonas aeruginosa* biofilms (Schooling and Beveridge, 2006) and more recently in biofilms formed by other species (Wang et al., 2015). OMVs are chemically heterogeneous multifunctional bilayer structures 50–250 nm in diameter, containing several macromolecules (i.e., phospholipids, proteins, lipopolysaccharide (LPS), and periplasmic components), which bleb from Gram-negative bacteria (Schooling and Beveridge, 2006; Mashburn-Warren et al., 2008). *In vitro* Transmission Electron Microscopy (TEM) analysis of biofilms showed highly variable OMVs densities within the matrix. Although, the biological role of OMVs has not been fully described, several studies have indicated that OMVs are involved in toxin and DNA transfer as well as in signaling between bacteria (Olofsson et al., 2010). Furthermore, OMVs can be integrated with membranes of other bacteria (Kadurugamuwa and Beveridge, 1999) or can be internalized into eukaryotic cells (Elluri et al., 2014; Jäger et al., 2014). *H. pylori* OMVs have previously been biochemically and functionally characterized by Olofsson et al. (2010), who demonstrated the presence of virulence factors involved in disease development as well as the adhesion proteins BabA and SabA, or the oncoprotein CagA, associated with the surface of OMVs. Moreover, there is evidence of *H. pylori* OMVs production *in vivo* (Fiocca et al., 1999; Heczko et al., 2000; Parker and Keenan, 2012).

Yonezawa et al. (2009) further demonstrated that the development of *H. pylori* biofilms depends on a direct cell-cell binding mediated by OMVs generated from bacteria, suggesting that OMVs serve as a “potentially novel gastric cell colonization factor of this organism.” We hypothesized that eDNA might associate with *H. pylori* OMVs in the biofilm to facilitate this cell to cell binding and contribute to the stability of the biofilm matrix. The goal of the present study was to detect and quantify the eDNA associated with OMVs isolated from biofilm and planktonic phenotypes of *H. pylori* ATCC 43629/NCTC 11639 by physically-chemically characterizing pOMVs and bOMVs, to elucidate a putative structural role of vesicles in the biofilm phenotype.

## Materials and methods

### Bacterial strains and media

The reference strain *H. pylori* ATCC 43629/NCTC 11639 was used in this study. Stocks were stored at −80°C before being thawed at room temperature, plated on Chocolate Agar (CA; Oxoid Limited, Hampshire, UK), supplemented with 1% (v/v) of IsoVitaleX (Becton Dickinson, Franklin Lakes, New Jersey, USA) and 10% (v/v) of defibrinated horse sterile blood (Oxoid Ltd), and finally incubated at 37°C for 3 days in a microaerophilic atmosphere (Campy Pak Jar; Oxoid Ltd; Drumm and Sherman, 1989).

### Biofilm formation assay

Bacteria were harvested in *Brucella* Broth (BB; Oxoid Ltd) supplemented with 2% (w/v) fetal calf serum (Sigma Aldrich, St. Louis, MO, USA) and 0.3% (w/v) of glucose (Grande et al., 2011). Broth cultures were incubated overnight at 37°C in a microaerophilic atmosphere under shaking at 90 rev min^−1^ (Innova 4300, New Brunswick Scientific, Edison, NJ, USA). After incubation, each broth culture was adjusted to an optical density at 600 nm (OD_600_) of 0.15 corresponding to 2.84 × 10^7^ CFU/ml and inoculated into 90 mm diameter Petri dishes (Corning Incorporated, New York, USA) and 35 mm diameter Petri dishes (Ibidi GmbH, Planegg, Germany). Bacteria were incubated at 37°C in a microaerophilic atmosphere, without shaking, for 48 h. After incubation, non-adherent cells were harvested, while the biofilms were rinsed with Phosphate Buffered Saline (PBS; pH 7.2). The biofilm cultures in 35 mm Petri dishes were used to test biofilm formation and cell viability by Live/Dead staining and Confocal Laser Scanning Microscopy (CLSM) analysis. The biofilm cultures in 90 mm Petri dishes were used for TEM analysis and OMVs extraction. Using a cell scraper, biofilms were scraped and transferred to 20 ml of PBS for further analysis. Broth cultures were plated on CA to ensure the absence of contaminating bacteria.

### Evaluation of biofilm formation and cell viability

*H. pylori* biofilms were grown as previously described (Grande et al., 2011) in 35 mm Petri dishes and examined for cell viability by confocal microscopy using the *BacLight* bacterial viability Kit (Life Technologies, Carlsbad, CA USA) according to the manufacturer's instructions. Briefly, the assay kit contains SYTO 9 and Propidium Iodide (PI) to evaluate cell membrane integrity, where green fluorescence indicates intact cells, red fluorescence dead or damaged cells. The samples were visualized using a Zeiss LSM510 META confocal system (Jena, Germany) connected to an inverted Zeiss Axiovert 200 microscope equipped with a Plan Neofluaroil-immersion objectives (63x/1.4 and 100x/1.45 NA). Fluorescent stains were excited using an argon laser beam with excitation lines at 488 nm (6% power) and a helium/neon 543 nm source (10% power), respectively. Primary and secondary dichroic mirrors, HTF 488/543 and NTF 545, were used to separate emission spectra of the two fluorochromes. The detector band-pass filters were set over 505–530 and 565–615 ranges for the green and red emission, which were alternatively recorded using the “MultiTrack” acquisition software to avoid overlap of fluorochrome emission. All experiments were performed at room temperature, and each Petri dish was examined for no longer than 10 min. Results are the average of three independent experiments, containing triplicate samples.

### OMVs extraction

The OMVs extraction from *H. pylori* was performed on biofilms as previously reported (Yonezawa et al., 2009) with slight modifications. Briefly, harvested *H. pylori* biofilms were centrifuged (5000 × g, 20 min at 4°C) and the resultant supernatants were filtered using 0.22 μm cellulose membrane filters (Corning, USA). Planktonic and biofilm filtrates (200 μl) were spread on CA and incubated at 37°C on microaerophilic conditions to confirm the total absence of viable *H. pylori*. The samples were further purified using a Beckman coulter Optima XL-100K ultracentrifuge (Beckman coulter, USA) at 50000 rpm, for 2 h at 4°C, washed with PBS and ultra-centrifuged for the second time (50000 rpm, 2 h at 4°C). The pellets were then resuspended in 200 μl PBS and stored both at −80°C and +4°C. The resultant planktonic (pOMVs) and biofilm (bOMVs) pellets were used for subsequent analysis.

### Physical-chemical characterization of pOMVs and bOMVs

#### OMVs sizing by dynamic light scattering (DLS)

DLS was used to characterize the size of the recovered OMVs as previously reported (Celia et al., 2013). Briefly, the OMVs were first filtered through a 0.22 μm cellulose filter membrane and further analyzed using a Zetasizer Nano ZS (Malvern Instruments Ltd., Worchestershire, United Kingdom), equipped with a 4.5 mW laser diode operating at 670 nm as a light source. The scattered photons were detected at 173°. A third-order cumulative fitting autocorrelation function was applied to determine the average sizes and size distribution. The analysis was carried out using the following instrument set up with a real refractive index of 1.59, an imaginary refractive index of 0.0, a medium refractive index of 1.330, and a medium viscosity of 1.0 mPa × s and a medium dielectric constant of 80.4 (Kirui et al., 2015). To avoid artifacts due to multiscattering, the pOMVs and bOMVs samples were suitably diluted before the analysis using pre-filtered (0.22 μm polypropylene membrane filter, Whatman Inc., Clifton, NJ, USA) RNAse free water. Results are reported as the average ± one standard deviation from 10 independent replicates.

#### Outer membrane charge of the OMVs by DLS

To measure the outer membrane charge and thus calculate the electrophoretic mobility, the Z-potential of the OMVs were performed using Doppler laser anemometry. A Smoluchowsky constant F (Ka) of 1.5 was applied during the analysis. The apparatus consisted of a He/Ne laser Doppler anemometry (633 nm) with a nominal power of 5.0 mW. Results are reported as the average ± one standard deviation from 10 independent replicates.

### Transmission electron microscopy (TEM) evaluation of eDNA associated with OMVs

To determine the association of eDNA-OMVs, DNase I-gold complex labeling of OMVs were visualized by TEM. The enzyme-gold complex was used to detect the location of DNA on planktonic and biofilm produced OMVs as previously reported (Bendayan, 1981). Briefly, biofilms were harvested after 48 h and the corresponding planktonic cultures, were centrifuged for 20 min at 4000 × g at 4°C, washed with PBS and processed to obtain ultrathin sections. Bacterial cells were fixed as previously reported (Tamburro et al., 2004) with slight modification. Cells were centrifuged and fixed with 2% (v/v) freshly prepared paraformaldehyde 0.1% (w/v) glutaraldehyde in 0.1 M cacodylate buffer pH 7.2, for 2 h at 4°C. Cells were washed thrice in the same buffer, pelleted, further dehydrated at scalar ethanol grade volume and embedded at 4°C in Unicryl Resin (BBI Solutions, UK) polymerized under UV irradiation.

To visualize pOMVs and bOMVs negative staining was performed. Formvar and carbon-coated copper grids (200 mesh; TAAB Lab. UK) were floated, film side down, on 20 μl of each sample for 20 s. Ultrathin sections were collected on nickel grids. The grids were first floated, for 5 min on a large drop of PBS, then the sections were incubated with DNase I-gold complex (E-Y Laboratories, San Mateo, CA, USA) diluted 1:10 (v/v) with 0.01M PBS (pH 6.0), containing 0.02% (v/v) polyethylene glycol (PEG) for 45 min, at room temperature. The grids were then thoroughly washed with PBS without PEG and rinsed in distilled water. In order to provide evidence of the amount of reaction, the grids were only stained with uranyl acetate [20 μl of 2% (w/v)] and gently touched to filter paper to remove excess sample/water. Controls were represented by samples treated without DNase I-gold and no gold particles were detected. The specimens were imaged using a transmission electron microscope Philips 268 D (FEI, Eindhoven, Netherlands) at 28000 times magnification. The number of bOMVs and pOMVs labeled with DNase I Complex were counted from the TEM images. Results are the average of three independent experiments, each with duplicate samples.

### eDNA detection and extraction

#### eDNA detection and quantification by PicoGreen staining

Extracellular DNA associated with pOMVs and bOMVs was detected in samples filtered through a 0.22 μm filter using the Quant-iT™ PicoGreen dsDNA assay kit (Life Technologies) according to manufacturer's instructions. From the same samples eDNA extraction was performed by using QIAamp DNA Mini Kit (Qiagen GmbH, Hilden, Germany) according to the manufacturer's protocol. Protein concentrations in *H. pylori* OMVs were quantified by using the bicinchoninic-acid (BCA) Protein Assay Kit (Pierce, Rockford, IL, USA) and bovine serum albumin (BSA) was used as protein standard to perform the calibration curve. 120 μg of proteins were extracted from each sample of pOMVs and bOMVs, respectively (Mincione et al., 2014) and the eDNA yield was normalized by using 2–5 μg of proteins.

The samples were prepared by mixing 100 μl of PicoGreen solution and OMVs made up to 200 μl in Tris-HCl buffer (10 mM, pH 8) and EDTA (1 mM; TE). The OMVs samples were incubated either with or without 1 U/μl DNase I (Sigma Aldrich) for 15 min at room temperature, then diluted and split into triplicate aliquots. A blank, consisting of 100 μl TE buffer with PicoGreen quantitation reagent, was used as control. PicoGreen staining was used to label the total dsDNA in different samples. DNase I further digested any external dsDNA not associated with the vesicles. Standard curves were constructed by serial dilution of the DNA stock solution provided in the kit. Samples were read in black microtiter plates (PerkinElmer, Waltham, MA USA) using a Glomax Multi Detection System (Promega, Madison WI, USA) at an excitation of 480 nm and emission wavelength of 520 nm. All measurements were carried out on triplicate aliquots each from a minimum of three independent experiments.

#### eDNA detection by agarose gel electrophoresis

DNA was extracted using QIAamp DNA Mini Kit (Qiagen) according to the manufacturer's protocol using the same samples analyzed as for the PicoGreen dsDNA assay. The yield of isolated DNA, untreated and treated with 1 U/μl DNase I (Sigma Aldrich), was determined from 1% (v/v) agarose gel electrophoresis by using Gel Doc EZ Imaging System with Image Lab Software (Bio-Rad Laboratories, Hercules, CA, USA). The results were independently verified by correlating to measurements made from a NanoDrop 2000c UV-Vis Spectrophotometer (Thermo Scientific, Rockford, IL, USA) and BioPhotometer (Eppendorf Hamburg, Germany) in order to assess accuracy of the PicoGreen data. The results were obtained from five independent experiments.

### Influence of exogenous DNA and subsequent digestion with DNase I on OMVs aggregation

DLS was also used to measure the size distribution of aggregated OMVs from planktonic and biofilm cultures and assess the influence of first adding exogenous DNA followed by adding DNaseI, to test our hypothesis that eDNA might play a role in aggregation and protection of eDNA from nuclease digestion. The aggregation of *H. pylori* OMVs was carried out by analyzing the polydispersity index (PDI) of samples. PDI was measured by using a Zetasizer Nano ZS (Malvern Instruments Ltd., Worchestershire, United Kingdom) and data were analyzed by using Zetasizer Nano ZS software (Malvern Instruments Ltd., Worchestershire, United Kingdom). A PDI from 0 to 0.25 indicates a narrow size distribution of *H. pylori* OMVs; while a PDI from 0.3 to 1 shows a broader distribution. Briefly, 2 μg of genomic DNA extracted from *H*. *pylori*, using QIAamp DNA Mini Kit (Qiagen), was added to 100 μl of pOMVs or bOMVs and further incubated at 37°C for 2 h in a water-bath. After incubation, the samples were incubated with DNase I for 15 min at room temperature according to manufacture's instruction. A control consisted of incubating samples with DNase buffer only. Results were obtained from triplicate samples of three independent experiments. The DLS aggregation studies were run as previously described in the “Physical-chemical Characterization of pOMVs and bOMVs” section.

### Statistical analysis

Results represent the mean of three experiments ± S.D (standard deviation) or SEM (standard error of the mean). Statistical analysis of data was performed using the *t*-test (Prism 3.0 GraphPad Software, San Diego, CA, USA). Differences were considered statistically significance for *p* ≤ 0.05.

## Results

### Biofilm characterization

**Figure 1:**
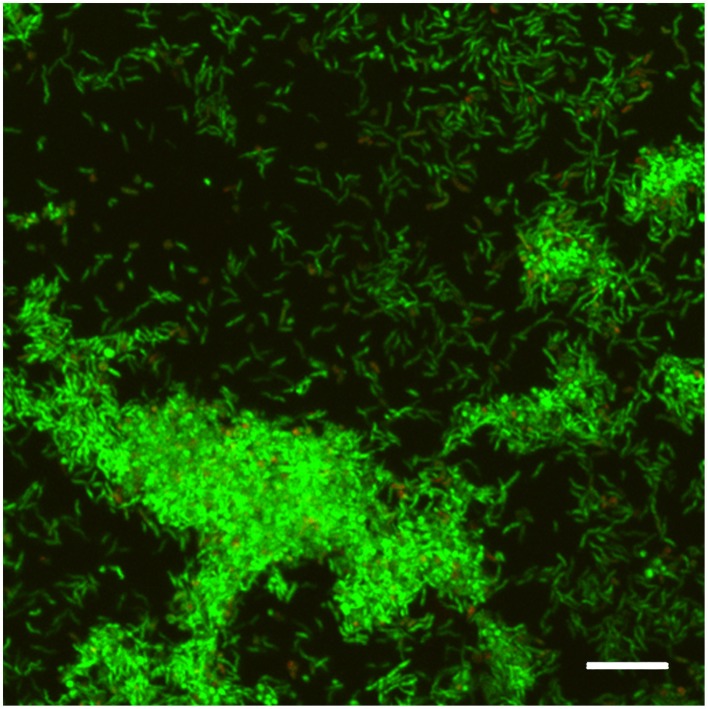
**CLSM representative image of biofilm obtained from ***H. pylori*** ATCC 43629/NCTC 11639 samples stained with SYTO 9 (viable cells, green fluorescence) and Propidium Iodide (dead cells, red fluorescence) after 48 h of incubation**. Scale bar = 5 μm. A representative image of five different experiments.

Biofilm formation was confirmed by Live/Dead staining and CLSM analysis. *H. pylori* ATCC 43629/NCTC 11639 48 h biofilms were relatively thin with an average thickness of 6–8 μm and showed 3D structure characterized by tower-like cell clusters heterogeneously interspersed with channels (Figure 1). Many single cells were attached to the surface in the interstitial channels. The biofilm consisted predominantly of live cells (green), while the number of dead cells (red) was negligible.

### Physical-chemical characterization of pOMVs and bOMVs

#### OMVs sizing by dynamic light scattering (DLS)

**Figure 2:**
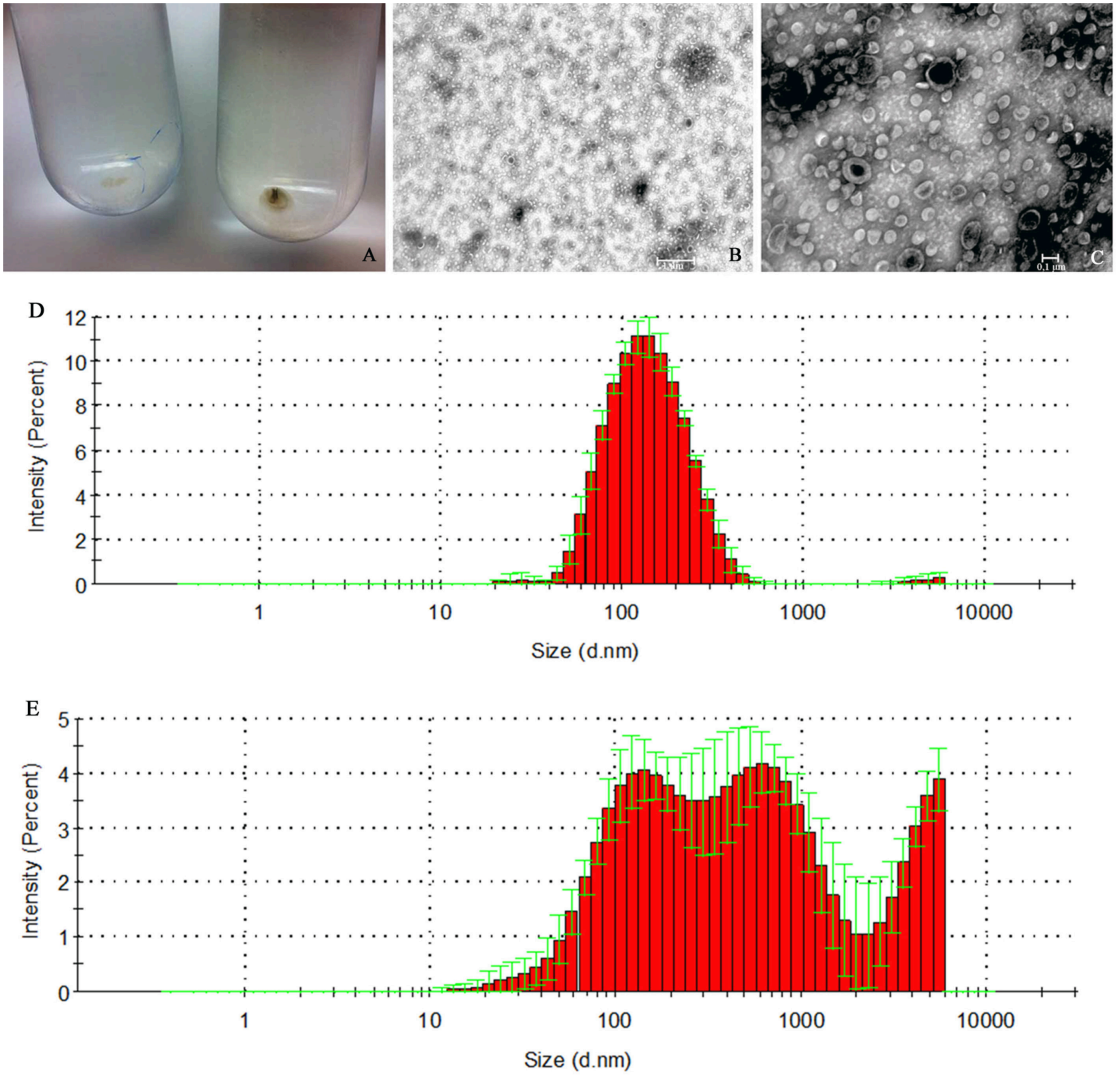
*****H. pylori*** ATCC 43629/NCTC 11639 pOMVs and bOMVs isolation**. **(A)** OMVs ultra-centrifuged pellets from *H. pylori* planktonic and biofilm phases. The bOMVs pellets (left) were smaller than the pOMVs (right) and were white in color while the pOMVs pellets were dark yellow. **(B,C)** Negative staining of vesicles generated by *H. pylori*. DLS histogram analysis of pOMVs and bOMVs are shown in panels **(D,E)** respectively. **(E)** bOMVs had a more heterogeneous bimodal distribution of nanovesicles. In fact, there are three peaks, the first one at 681.1 nm (S.D. ± 256.2), the second one at 1760 nm (S.D. ± 2216) and the third one at 2557 nm. Figures are representative of five measurements.

**Figure 3:**
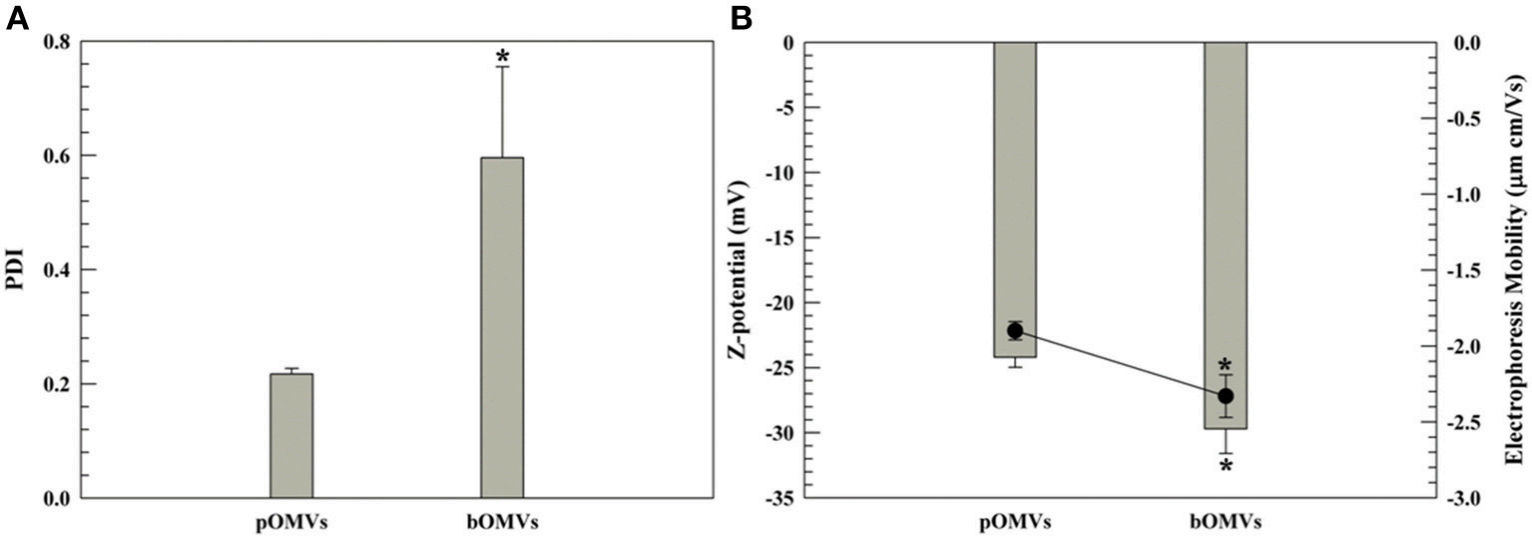
**DLS panel (PDI and Z-potential)**. PDI **(A)** and Z-potential **(B)** of pOMVs and bOMVs. **(B)**, the histograms represent the Z-potential values (mV); while the black full circles are the electrophoretic mobility [(μm × cm)/Vs]. The sample analysis was carried out at 25°C and data are the average of 10 measurements ± standard deviation as triplicates. The error bar if not visible is within the symbol. ^*^p ≤ 0.05.

The pOMVs were spherical in shape with an average diameter of 123.7 nm (S.D. ± 1.6; Figure 2), and a size distribution (PDI) of 0.217 (S.D. ± 0.01; Figure 3A). Conversely, the bOMVs show a broader distribution of particle sizes ranging from 10 nm to 2.5 μm with a median value of 1760 nm (S.D. ± 1135.84) and an average value of 293.1 nm (S.D. ± 38.68). In particular, bOMVs showed three main peaks with different percentages of particles distributed at 681.1 nm (Peak 1; 59%), 1760 nm (Peak 2; 29.9%) and 2557 (Peak 3; 11%), respectively (Figure 2). The broad particle distribution of bOMVs was further supported from PDI, which yielded a value of 0.596 (S.D. ± 0.159; Figure 3) suggesting heterogeneous bOMVs aggregation in different samples. The average size and size distribution of both OMVs generally agreed with the TEM analysis, however the OMV could not be directly measured since the thin sections did not necessarily slice the particles through the diameter (Figure 2). Larger particles (906 nm, S.D. ± 2010; 1%) may be aggregates forming after the filtration though the 0.22 μm polypropylene membrane filter; while smaller particles (98.8 nm, S.D. ± 1.7; 0.2%) may be small fragment of particles.

#### Outer membrane charge of the OMVs by DLS

The Z-potential and electrophoresis mobility of pOMVs and bOMVs showed negative values for both phenotypes consistent with a cell wall charge. The pOMVs had a Z-potential of −25 mV and an electrophoretic mobility of −1.9 (μm × cm)/Vs, respectively. The Z-potential of the bOMVs was −30 mV with an electrophoretic mobility of −2.5 (μm × cm)/Vs, respectively (Figure 3B). The electrophoretic mobility values, which are measured concomitantly by the Zetasizer Nano ZS instrument (Malvern) were used to corroborate the Z-potential values, as has been previously described (Wolfram et al., 2014). The Z-potential analysis of pOMVs and bOMVs demonstrated that a net negative charge surrounded the OMVs on their outer surface, consistent with a cell wall charge. The Z-potential of bOMVs was significantly different (*p* ≤ 0.05) from pOMVs. The Z-potential of OMVs was more negative compared with the Z-potential of genomic DNA extracted from *H. pylori*, which was −16.07 mV (S.D. ± 0.76; Supplementary Figure 1). LPS and divalent cations or salt bridges, characteristic of Gram-negative bacteria, may further affect the net negative charge of *H. pylori* OMVs (Kadurugamuwa and Beveridge, 1995, 1996).

### eDNA detection in pOMVs and bOMVs by TEM and DNase I-Gold complex labeling

**Figure 4:**
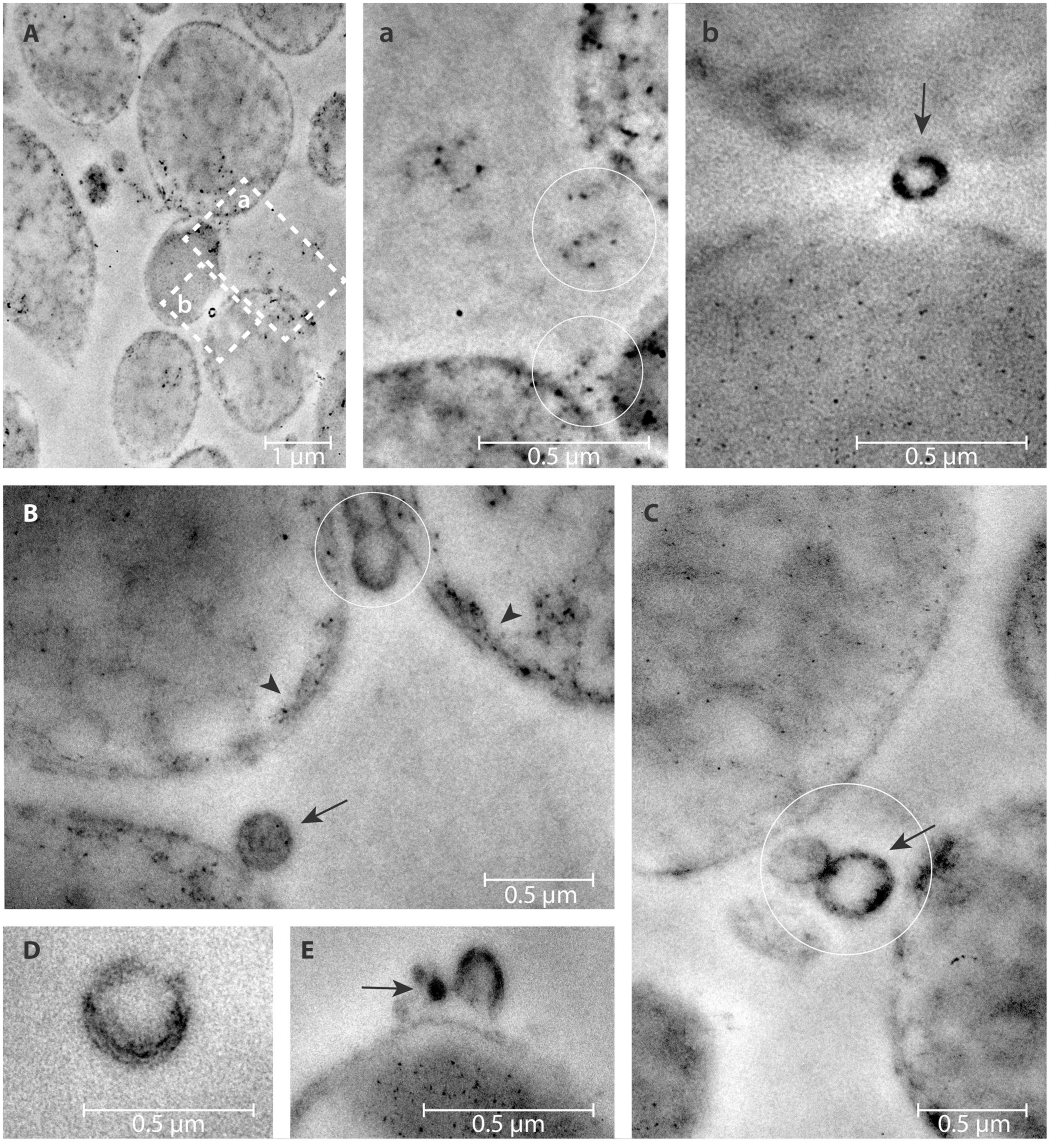
**DNase I-gold complexes labeling of 2-days old ***H. pylori*** ATCC 43629/NCTC 11639 mature biofilms**. The colloidal gold labeled DNase I particles are used to detect the eDNA. The arrow represents the presence of eDNA on bOMVs membrane surface **(b,B,C,E)**; while the arrow head represents the presence of eDNA on bacteria cell walls **(B)**. OMVs are present between adjacent bacterial cells (circles, **a**,**B**,**C**). Representative arrangements between cells and vesicles are shown at higher resolution by the dashed squares “**a**” and “**b**” in **(A)**. Panel **(D)** shows a single vesicle labeled with colloidal gold labeled DNase I particles.

**Figure 5:**
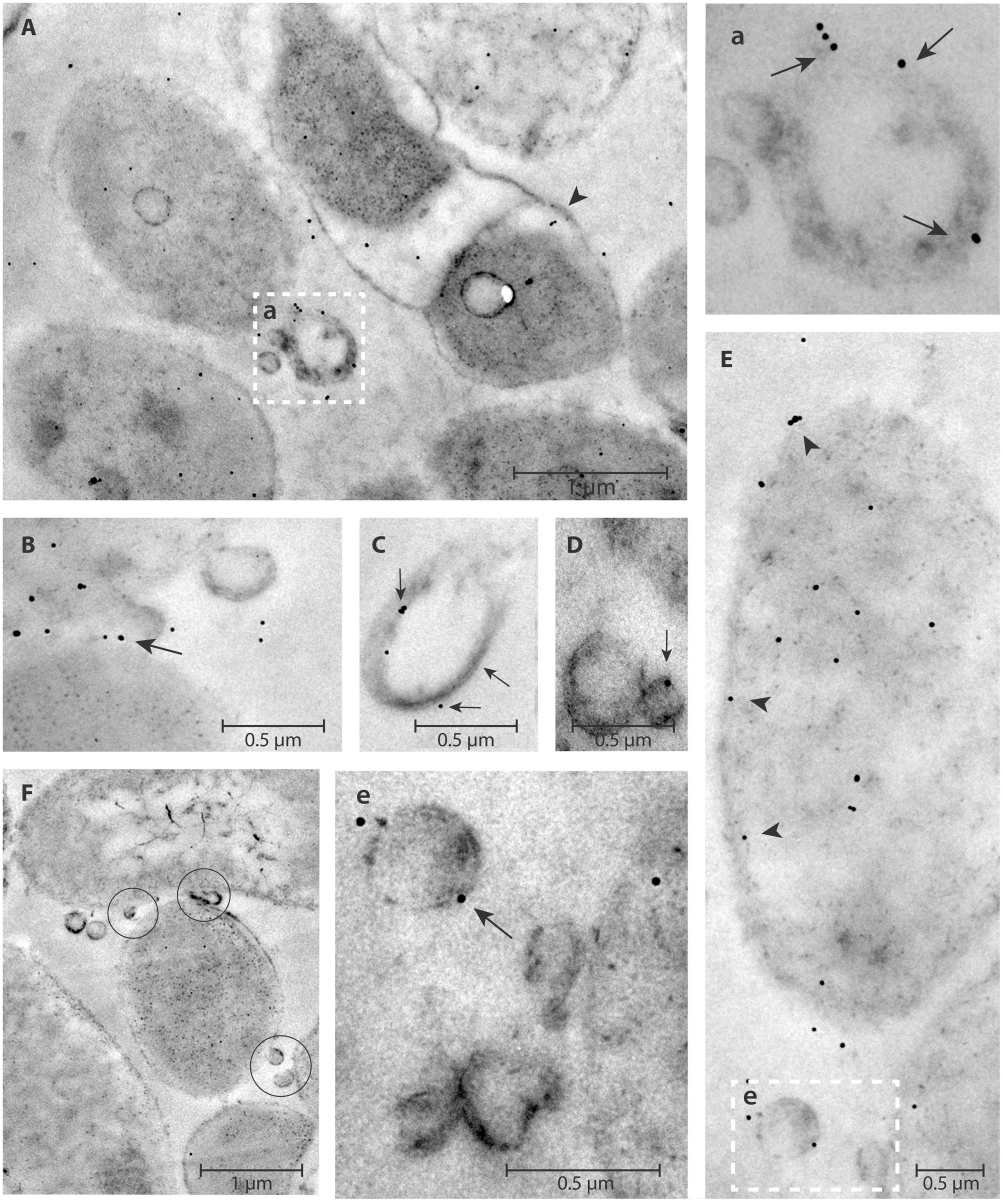
**DNase I-gold complexes labeling of ***H. pylori*** ATCC 43629/NCTC 11639 planktonic phase**. The colloidal gold labeled DNase I particles are used to detect the eDNA. The arrow represents the presence of eDNA on pOMVs membrane surface **(a,B,C,D,e)**; while the arrow head **(A,E)** represents the presence of eDNA on bacteria cell walls. OMVs appear between two cells (**F**, circles). The dashed squares **(a,e)**, higher magnification of **(A,E)**, highlight eDNA on the pOMVs surface.

eDNA associated with both bOMVs and pOMVs was localized mainly on the surface of vesicles (Figures 4a,b,B–E, 5A,a,B–E,e,F). The percent of vesicles labeled with DNase I-gold was 30% for bOMVs, which was significantly more than the 12% for pOMVs (*p* ≤ 0.05; Table 1). The presence of DNA was also detected on quasi-spherical blebs originating from the cell wall, which appeared to be OMVs in the process of shedding (Figures 4B, 5F). TEM images also showed aggregates of OMVs (Figure 4C) and bacterial cell aggregation (Figures 4A,a,B). More DNA was associated with bOMVs than pOMVs, and bOMVs appeared to aggregate with other OMVs and other cells, suggesting that the eDNA might play a bridging role (Figures 4A,a,b,B,C). Aggregates were more common in the biofilm phenotype. Any non-specific DNase I-gold was detected in the background.

**Table 1:** **Percentage of OMVs labeled with DNase I-gold complexes to detect the presence of eDNA on the OMVs surface**.

**OMVs**	**Number of OMVs (%)**
bOMVs	30.0 ± 3.87
pOMVs	12.0 ± 2.7

*The analysis was carried out analyzing 30 pOMVs and bOMVs collected from TEM images of five experiments (n = 5) and data is reported as a percentage of total OMVs counting (average ± S.E.M.)*.

### eDNA detection and extraction

#### eDNA detection and quantification by PicoGreen staining

**Figure 6:**
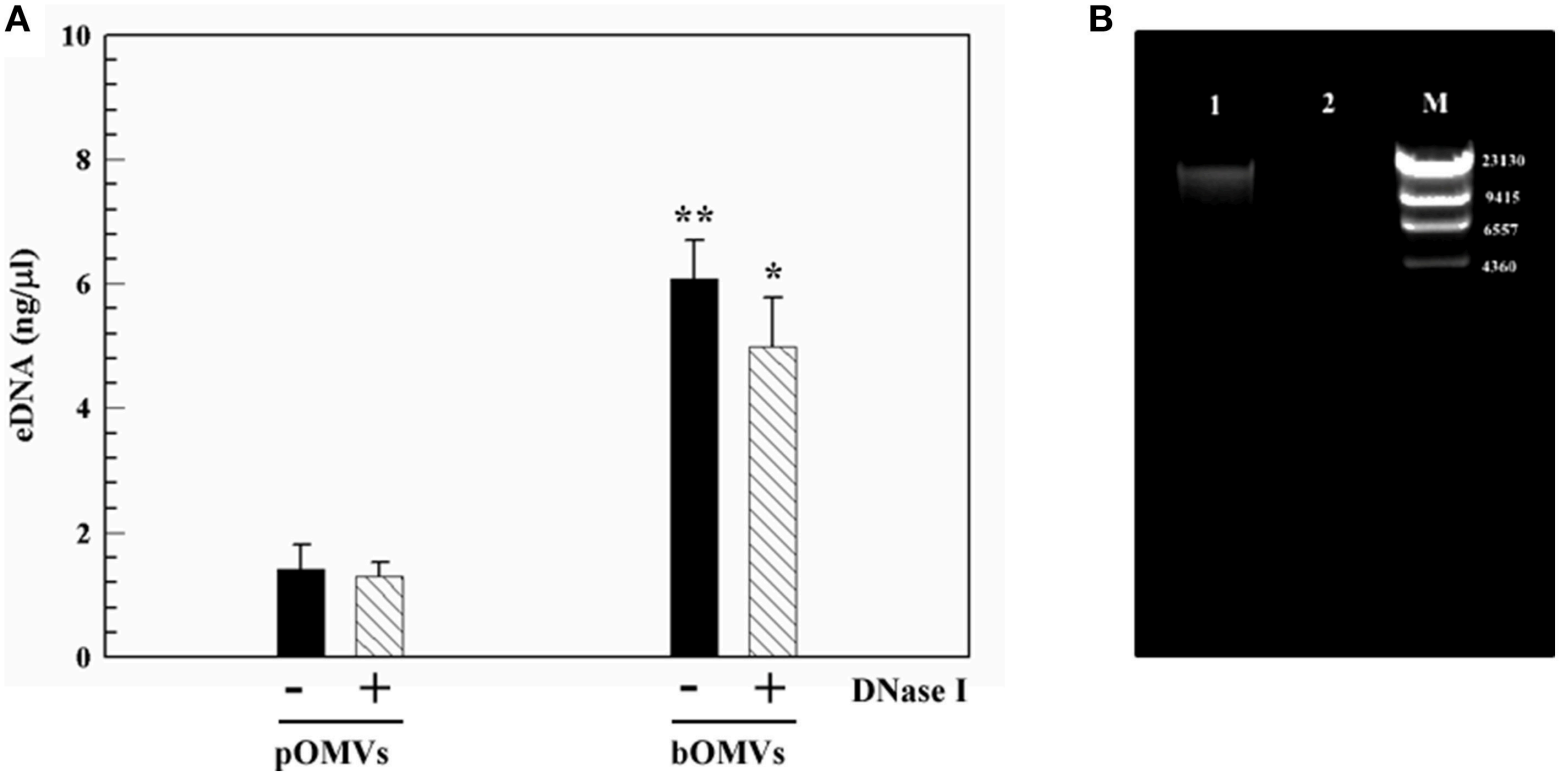
**eDNA associated with OMVs**. **(A)** Detection and quantification of eDNA-pOMVs and eDNA-bOMVs associated, DNase I treated and untreated, by using PicoGreen assay. ^**^p ≤ 0.003; ^*^p ≤ 0.010 for differences of bOMVs compared to pOMVs. The protein content was used to normalize eDNA. **(B)** Representative image of agarose gel analysis of bOMVs-DNA from *H. pylori* ATCC 43629/NCTC 11639, untreated (lane 1), and DNase I-treated (lane 2). The lambda DNA/HindIII marker (M), DNA fragments are in base pairs.

The concentration of DNA associated with pOMVs and bOMVs samples was quantified directly using PicoGreen staining in a micro-plate assay. PicoGreen labeled the total dsDNA in the samples and the addition of DNase I digested the unbound “free” eDNA, thus facilitating PicoGreen labeling of DNA associated with OMVs. dsDNA was associated with intact OMVs from planktonic and biofilm phenotypes in both untreated and DNase I-treated samples (Figure 6A). However, the eDNA content in OMVs isolated from the 48 h biofilm was four-fold greater than eDNA associated with OMVs isolated by planktonic cultures of the same age and normalized by protein content.

#### eDNA detection by agarose gel electrophoresis

To independently corroborate the PicoGreen staining microplate assay results, DNA extracted from bOMVs samples, untreated and treated with DNase I, was analyzed by agarose gel electrophoresis. DNA was detected in association with vesicles (Figure 6B, lane 1), however it was degraded after DNase I-treatment (Figure 6B, lane 2). The extracted DNA had a molecular weight greater than 9415 base pairs (bp).

### Influence of exogenous DNA and subsequent digestion with DNase I on OMVs aggregation

When exogenous DNA was added to pOMVs or bOMVs, the average aggregate diameter increased from 123.7 nm (S.D. ± 1.6) to 147.5 nm (S.D. ± 2.4) for pOMVs, and 293.1 nm (S.D. ± 38.68) to 890.7 nm (S.D. 135) for bOMVs. The treated bOMVs had a median value of 2369.3 nm (S.D. ± 1232.75), and the DNA aggregation distribution of DNAse treated bOMVs was bimodal with two main peaks at 447.6 nm (S.D. ± 97.75) and 4291 nm (S.D. ± 2368). Non-treated bOMVs showed a median value of 1760 (S.D. ± 2216) and had a multimodal distribution with a peak at 681.1 nm (S.D. ± 256.2), 1760 nm (S.D. ± 2216) and 2557 nm (S.D. ± 2331), respectively (Supplementary Figure 2). Conversely, the particle sizes of pOMVs, exposed to exogenous DNA and followed by treatment with DNase I, were similar to native pOMVs (Supplementary Figure 3). In fact, the treated pOMVs showed a median value of 1952 nm (S.D. ± 2169), and the DNA aggregation distribution of DNAse-treated pOMVs was multimodal with three main peaks at 171.8 nm (S.D. ± 34.70), 3151 nm (S.D. ± 2169), and 1952 nm (S.D. ± 2676); whereas non-treated pOMVs had a modal distribution with a peak at 152.6 nm (S.D. ± 4.32) for 98% of particles. The secondary peak (1% of particles) and third peak (0.2 of particles) of pOMVs are 906 nm (S.D. ± 2010) and 938.4 nm (S.D. ± 2199), respectively (Figure 2). These results, indicating increased aggregation of bOMVs by adding exogenous DNA and treating with DNase I, were further supported by PDI, which increased from 0.596 to 0.724.

## Discussion

*H. pylori* produces OMVs in both planktonic (Olofsson et al., 2010) and biofilm (Yonezawa et al., 2009) phenotypes. However, pOMVs contain different phospholipid fractions compared with bOMVs (e.g., phosphatidylethanolamine, lysophosphatidylethanolamine, cardiolipin, phosphatidyl-glycerol, and cholesterol), and proteins, (e.g., CagA, BabA, and VacA), which can affect virulence and pathogenicity (Olofsson et al., 2010). To date few papers discuss the characteristics of bOMVs released from *H. pylori* (Yonezawa et al., 2009), despite the fact that Yonezawa et al. (2011) demonstrated that OMVs produced from the clinical strain *H. pylori* TK1402 played an important role in biofilm formation. To better characterize the OMVs produced by *H. pylori* growing as biofilms and compare them with OMVs produced by planktonically-growing cells, we performed size and aggregation analysis using dynamic light scattering (DLS). DLS analysis indicated that pOMVs and bOMVs had different peak widths, but suggested that both OMVs have a multilayer form. The different peaks suggest the first- and second- order diffraction of a lamellar bilayer and thus are related to the modification of bilayer asymmetric structure as has been previously demonstrated for bacteria (Jäger et al., 2014). Differences in the average size and size distribution of pOMVs and bOMVs may be dependent on the temperature-dependent liquid to crystalline phase transition of lipids forming the OMVs. The transition from crystalline to liquid phase can increase the fluidity of OMVs and promote the fusion between natural vesicles, thus changing both their average sizes and size distribution (Jäger et al., 2014). The presence of phospholipase C in the microenvironment can degrade phospholipids forming OMVs and change the size distribution and original structure of nanovesicles generated from bacteria (Chebotar et al., 2013).

eDNA associated with OMVs was demonstrated by PicoGreen and TEM. DNase I-gold labeling of dsDNA indicated that it formed a complex on the outer layer of *H. pylori*. The significant accumulation of eDNA associated wtih bOMVs suggests a different role of bOMVs compared with pOMVs. bOMVs were more aggregated than pOMVs, which may depend on the different physical interaction between OMVs due to the greater amount of eDNA on their surface. The accumulation of eDNA on the outer layer of OMVs and the microscopic evidence of aggregation suggests eDNA may play a role in “bridging” OMV-OMV and OMV-cell interactions and supports the hypothesis that eDNA plays an important role in biofilm formation as previously demonstrated (Yonezawa et al., 2009). Our data further suggested that intact vesicles with internal eDNA blebbed from both the planktonic and biofilm phenotype of *H. pylori* and may prevent nucleic acid degradation after its release from the bacteria membrane. These data are in agreement with results from Schooling et al. (2009), who demonstrated the presence of DNA associated with the lumen and external face of vesicles generated by *Pseudomonas aeruginosa* PAO1. Z-potential analysis showed a net negative charge of OMVs and that bOMVs had a greater negative charge than pOMVs. This effect may be explained by differing amount of the eDNA located on the external surface of OMVs. The net negative charge of bOMVs might further affect their potential interactions with biofilm EPS components, as well as with cells, and might affect the mechanism of action of antibiotics (Chebotar et al., 2013; Elluri et al., 2014).

Previously we demonstrated that the EPS matrix of *H. pylori* biofilm contained eDNA (Grande et al., 2011). The ineffectiveness of DNase I, added both during biofilm formation, and to 48 h biofilms, suggested that eDNA was stabilized and/or protected by other EPS components, preventing degradation/disassembly of the biofilm biomass, as has been previously demonstrated for other microorganisms (Grande et al., 2014). Here we report that OMVs in the biofilm may protect eDNA. The content of dsDNA was four-fold higher in bOMVs than in pOMVs, suggesting that, in addition to being a structural component of the biofilm, DNA might also has a role in aggregation. These results are similar to those for *Enterococcus faecalis* biofilms, where eDNA increased compared with planktonic cultures (Barnes et al., 2012). Recently, Liao et al. (2014) showed that eDNA blebs from *Streptococcus mutans* were actively released from bacteria and were independent of cell lysis.

Importantly, no significant difference in eDNA concentration was detected between DNase I-treated and untreated pOMVs and bOMVs samples, suggesting that the nucleic acid was associated with OMVs and protected from nuclease digestion. Although it is not yet clear whether the association of OMVs with eDNA or *H. pylori* cell walls plays a functional role, we speculate that DNA associated with OMVs might have a role in DNA transfer in both planktonic and biofilm phenotypes and additionally in the structural integrity of *H. pylori* biofilms. These hypotheses will be further investigated in future work to elucidate the fate of eDNA contained in pOMVs and bOMVs, before and after interaction with bacteria and gastric epithelial cells, as well as sequencing the dsDNA that is being transported.

In conclusion, differences in eDNA associated with pOMVs and bOMVs, suggest potentially different roles for planktonic and biofilm growth of *H. pylori* in infection and disease and demonstrate the potential of biofilms to act as a reserve for genetic material. The physical-chemical evaluation of OMVs indicated that bacteria could generate native nanovesicles containing DNA, which may have clinical relevance. Furthermore, nanovesicles may be useful for the development of anti-biofilm pharmacological agents for *H. pylori*. OMVs generated from planktonic and biofilm *H. pylori* may play a role in the development of biofilm infection and therefore understanding the biological activity and composition of these structures may be important for better understanding the pathogenesis of this bacterium.

## Author contributions

RG designed the project, isolated pOMVs and bOMVs from *H. pylori*, discussed results and drafted the paper. MM, GM, and RM performed the PicoGreen experiments, the DNA extraction, analyzed *in vitro* data, discussed results and drafted the PicoGreen figures. GM wrote the paper together with RG. IR performed the eDNA detection in pOMVs and bOMVs by DNaseI-labeled complex, discussed results and drafted TEM images. PS, RG, and CC performed the OMVs aggregation assay. CC, LM, and DP performed the DLS aggregation analysis of pOMVs and bOMVs, discussed results and drafted the DLS figures. CC and PS discussed DLS aggregation data and drafted the DLS aggregation figures. AP performed CLSM analysis. MC provided technical assistance to ultra-centrifuge. PS, LS, RG, CC drafted the final editing of paper and critical revised paper.

### Conflict of interest statement

The authors declare that the research was conducted in the absence of any commercial or financial relationships that could be construed as a potential conflict of interest.
