# Supplementary material for: Helicobacter pylori ATCC 43629/NCTC 11639 Outer Membrane Vesicles (OMVs) from Biofilm and Planktonic Phase Associated with Extracellular DNA (eDNA)
Source: Front Microbiol. 2015 Dec 16;6:1369. doi: 10.3389/fmicb.2015.01369 (PMC4679919; doi:10.3389/fmicb.2015.01369)
Supplement: Supplementary file 1 [file DataSheet4.pdf]

Helicobacter pylori **ATCC 43629/NCTC 11639 outer membrane vesicles (OMVs) from biofilm and planktonic phase associated with extracellular DNA (eDNA).**

Rossella Grande[1,2,*], Maria Carmela Di Marcantonio[2,3,†], Iole Robuffo[4,†] , Arianna Pompilio[2,3], Christian Celia[1,5], Luisa Di Marzio[1], Donatella Paolino[6], Marilina Codagnone[2,3], Raffaella Muraro[2,3], Paul Stoodley[7,8,9], Luanne Hall-Stoodley[7,10], Gabriella Mincione[2,3].

[1]Department of Pharmacy, University of Chieti-Pescara "G. d'Annunzio", Chieti, Italy, [2]Center of Excellence on Aging, Ce.S.I., University of Chieti-Pescara "G. d'Annunzio", Chieti, Italy, [3]Department of Medical, Oral, and Biotechnological Sciences, University of Chieti-Pescara "G. d'Annunzio", Chieti, Italy, [4]Department of Biological Science, Institute of Molecular Genetics, National Research Council (CNR), Chieti, Italy, [5]Department of Nanomedicine, Houston Methodist Research Institute, Houston, TX, 77030, USA, [6]Department of Clinical and Experimental Medicine, University of Catanzaro "Magna Graecia", Germaneto – Catanzaro, Italy, [7]Department of Microbial Infection and Immunity, Center for Microbial Interface Biology, The Ohio State University, Columbus, OH, USA, [8]Department of Orthopaedics, The Ohio State University, Columbus, OH, USA, [9]Faculty of Engineering and the Environment, University of Southampton, UK, [10]NIHR Wellcome Trust Clinical Research Facility, University Hospital Southampton NHS Foundation Trust, Southampton, UK.

†MCDM and IR contributed equally to this work.

*Correspondence:

Dr. Rossella Grande
University of Chieti-Pescara "G. d'Annunzio",
Department of Pharmacy,
Via dei Vestini 31,
66100 Chieti, Italy.
E-mail: r.grande@unich.it

Running title: eDNA in *H. pylori* OMVs.

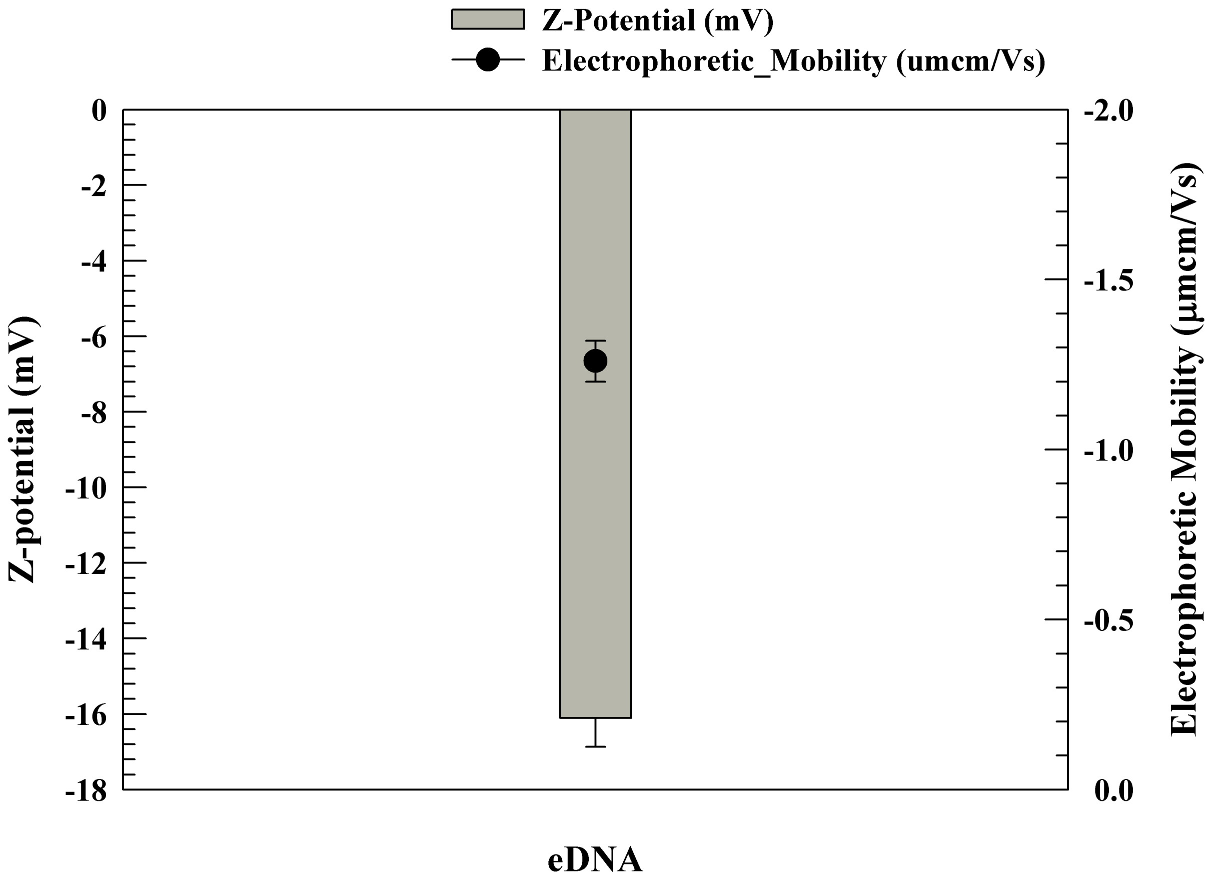

SUPPLEMENTARY FIGURE 1 | Z-potential and electrophoretic mobility of naked exogeneous DNA in PBS buffer used to disperse *H. pylori* OMVs. The analysis was carried out using DLS at 25°C. 3 µg/100 ml of samples was used during the analysis. Measurements are ten run of single sample as replicates (n = 3).

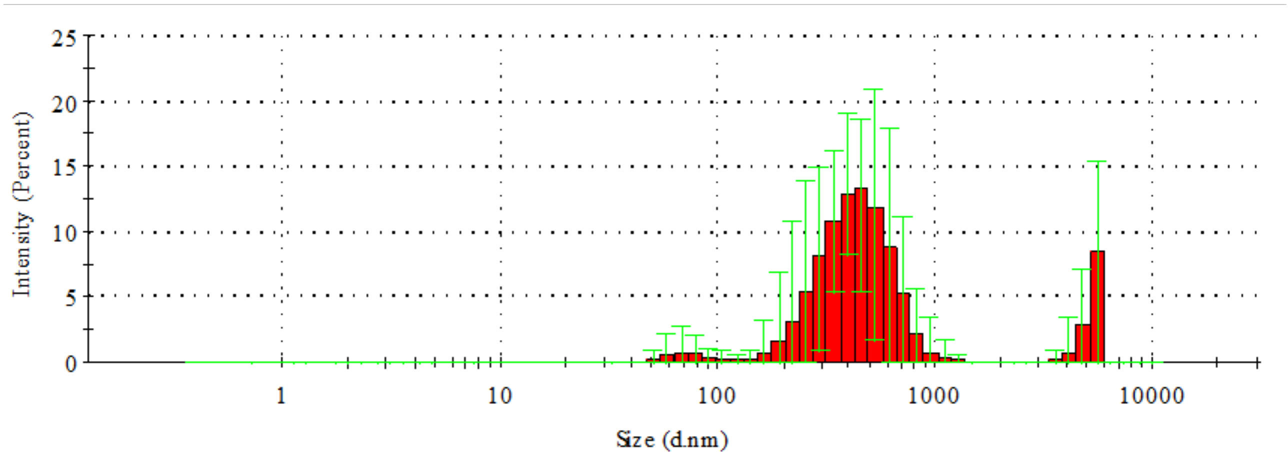

SUPPLEMENTARY FIGURE 2 | The DLS analysis of *H. pylori* bOMVs by adding exogenous DNA and its following digestion in DNaseI at 25°C. The histogram represents the aggregation of *H. pylori* bOMVs. The broad distribution (PDI = 0.724, S.D. ± 0.11) of *H. pylori* bOMVs depends on sample aggregation. Figures are representative of five measurements (intensity versus size (nm)), which are exported after DLS analysis using the Malvern Zetasizer NanoZS software.

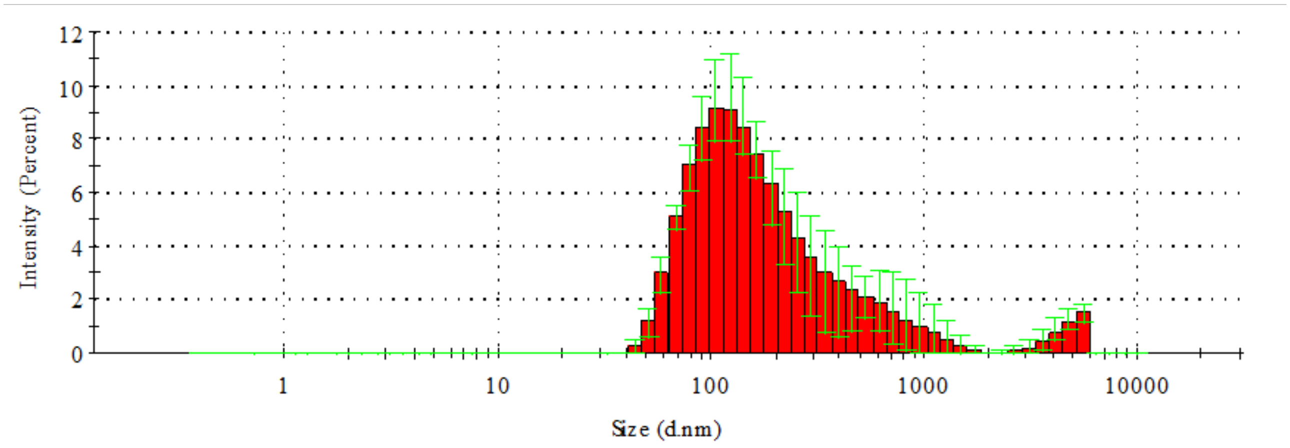

SUPPLEMENTARY FIGURE 3 | The DLS analysis of *H. pylori* pOMVs by adding exogenous DNA and its following digestion in DNaseI at 25°C. The histogram represents the aggregation of *H. pylori* pOMVs. The broad distribution (PDI = 0.453, S.D. ± 0.1) of *H. pylori* pOMVs depends on sample aggregation. Figures are representative of five measurements (intensity versus size (nm)), which are exported after DLS analysis using the Malvern Zetasizer NanoZS software.
